# 

**Published:** 1916-06-24

**Authors:** 


### RECENT HISTORY AND SERBIAN AIMS.

A book which will shortly appear (J. and A. Churchill) is written by members of a Red Cross unit in Serbia. The volume is, by permission, dedicated to the Crown Prince of Serbia, and contains his photograph and other illustrations.

### Gifts to Hospitals.

TUNBRIDGE WELLS GENERAL HOSPITAL receives £1,000 under the will of Miss Annie Emily Pitman.

THE KING has contributed £100 to the Lancashire Military Convalescent Hospital at Blackpool.

UNDER the will of Lord Armitstead the Royal Infirmary, Dundee, has become entitled to £10,000.

MRS. CONSTANCE C. WOOD, of Yateley, Hants, left £100 to the Royal Hospital for Incurables, Putney.

IN memory of Lord Kitchener, Sir William James Thomas has given a thousand guineas to Cardiff Hospital to endow a bed.

MANCHESTER INFIRMARY and Southport Infirmary each receive a sum of approximately £2,000 under the will of Miss Charlotte Emily Beckwith, of Southport, who died on March 7.

THE Royal Dental Hospital, Leicester Square, has received a donation of £400 from the trustees of Smith's (Kensington Estate) Charity, and £100 from the executors of the late Mr. William Johnston.

IN addition to bequests of £100 each to the Bolton Infirmary and the Samaritan Hospital for Women and Children, Alderman William Nicholson, of Bolton, left two-thirds of his residuary estate to the Northern Counties Supplementary Hospital for Chronic and Incurable Diseases.

MISS MARION E. O. WOOD, of Old Trafford, Manchester, has presented £1,000 to Ancoats Hospital, Manchester, to endow a bed in memory of her brother, the late Dr. J. W. A. Wood. This hospital has also received £500 from Mr. J. Robertshaw, of Ancoats, Manchester, to endow the "Robertshaw Golden Wedding Memorial Cot."

### PASSING EVENTS AND TOPICS.

### A Gift from India.

Mrs. Stella Rogers has collected 17,000 rupees (£1,138 17s. 3d.) in the district of Pegu, Burma, in aid of the funds of the Queen Mary's Convalescent Auxiliary Hospitals, Roehampton.

### A Nottingham Motor-Ambulance.

The Federation of Lace and Embroidery Employees' Associations has launched a movement for providing the funds to purchase, equip, and maintain, in the name of the lace trade, at least one motor-ambulance for the use of the Red Cross Society.

### The Work of a War Hospitals Supply Centre.

During one month the Central Store for Surgical Dressings at Glasgow dispatched 31,834 bandages, dressings, and splints to ambulances, casualty clearing stations, hospitals, ambulance trains, and hospital ships abroad and to home hospitals, along with 582 bought packets of lint, wool, etc.

### Convalescent Camps after the War.

Opening a gymnasium and concert hall at the King's Military Hospital, Blackpool, erected at a cost of £2,200 by subscriptions from Lancashire people, Lieutenant-Colonel Baron, the officer commanding, said he had wondered what would become of the hospital after the war. He suggested to the Corporation that if they could possibly see their way they might transform it into a camp for the physical training of the poorer children of Lancashire.

### A Cavell Memorial.

The proposal to establish a Home of Rest for Nurses as a memorial to Miss Cavell has received a considerable fillip by the offer of a house and garden at Hindhead, valued at £2,500. A sum of £10,000, it is stated, will be necessary for an endowment fund. It is not easy to imagine any district better suited for this purpose than Hindhead; so it may be hoped that the endowment necessary will be forthcoming.

### Medicine for Dependants of Sailors and Soldiers.

Under the scheme organised by the British Medical Association and the Pharmaceutical Society for providing medical advice and medicines free of charge for the necessitous dependants of sailors and soldiers, some million and a quarter prescriptions have already been distributed. Pharmacists make no charge for dispensing these, but receive the actual cost of the drugs used from the Prince of Wales' Fund. It is estimated that if each pharmacist had been remunerated for his services as a dispenser on the basis of the Insurance Act tariff the fees would have amounted in the aggregate to £13,000.

### WHAT'S WHAT COLUMN.

[*To meet the wants and wishes of many readers we have organised this Column. Its usefulness must depend upon its continuous use by Matrons, Sisters, and hospital workers. If full opportunity is taken of this Column the result may prove of world-wide importance.*]

### ANSWERS TO CORRESPONDENTS.

QUEEN'S, VILLAGE, AND COTTAGE NURSES?

*B. F.*—Can you tell me what is the difference, if any, between a Queen's nurse, a village nurse, and a cottage nurse?

There is all the difference in the world between a Queen's nurse and a village nurse. A Queen's has a three-years' certificate from a recognised training-school, and six months' special training under a trained, experienced superintendent in district nursing, and many Queen's nurses also have to possess their C.M.B. and school nurse's certificates. A village nurse has had no hospital training at all, but twelve months' district training only, and during this time she is also trained as a midwife. Both Queen's and village nurses work on the visiting system. A cottage nurse has had three to six months' district training. A small percentage have had midwifery training, but the majority are maternity nurses only. These women live in the cottage where their patient resides and look after the children and house while the mother is laid up. Many people think that a better name for these women would be cottage helps, and as such they would fill a great need.

THE STAFF OF A COTTAGE HOSPITAL?

*Matron C. H.*—I have been appointed as matron of a newly built cottage hospital (thirty-five beds). The hospital is near a large factory, and the work is expected to be acute. Could you advise me how best to get a staff, and what number I should require, and what salaries should be given?

Your best way to get your trained staff will be to advertise, and write to any matrons of training-schools you know. You would probably require two sisters—one for night and one for day—and one staff nurse for day. If you make a point of having all the labour-saving appliances you can get, one wardmaid, one cook, and one housemaid should be enough, with possibly an occasional charwoman. If you want good, well-trained, experienced workers you must offer good salaries. I suggest for the two sisters £50 each, rising by £5 to £60; the staff nurse, £40, rising to £50; the cook, a most important person, £40 to £45; the other two maids, £18 to £24; "all found" in each case. I have not mentioned probationers, as unless you could combine the training you could give with a larger hospital or infirmary, it would not be fair to take a pupil-nurse. You would find it a great help if you wrote in a book for each worker exactly what her work is, hours on and off duty, and meal hours.

### Medical Appointments.

THE directors of the Glasgow Ear, Nose, and Throat Hospital have appointed Mr. P. T. Young, LL.B., of 93 West Regent Street, Glasgow, to the post of secretary and acting treasurer, rendered vacant by the death of Mr. Adam Sutherland.

DR. PARSONS, of the Birmingham General Hospital, has been appointed principal medical officer of the hospital unit recently organised in Birmingham for service in the East. The unit is at Aldershot at present.

MR. B. J. RODWAY, L.D.S. (Eng.), who has been appointed lecturer in Dental Mechanics and Dental Prosthetics at Manchester University, has also been appointed by the War Office honorary consultant in dental surgery for face and jaw injuries at the Second Western General Hospital, Manchester.

### Matrons' and Sisters' Appointments.

BECKENHAM COTTAGE HOSPITAL.—Miss I. Allen has been appointed matron. She was trained at Guy's Hospital, where she was later staff nurse and head nurse in charge of a surgical ward. She has since been matron of Mold Cottage Hospital and sister in charge of the training-school, Queen Charlotte's Hospital.

HARBORNE HALL AUXILIARY HOSPITAL, BIRMINGHAM.—Miss Olive Langhorn has been appointed matron. She was trained at Manchester Royal Infirmary, where she was later temporary sister, and has also been sister at the County of London War Hospital, Epsom.

WIGAN INFECTIOUS DISEASES HOSPITAL.—Miss Mary Moss has been appointed matron. She was trained at Manchester Royal Infirmary, where she later became charge nurse, and at Monsall Fever Hospital. Miss Moss has also been matron of Stockport Isolation Hospital, Ramsey Isolation Hospital, and Ashton-under-Lyne Borough Hospital.

BEDFORD UNION INFIRMARY.—Miss Mary A. Price has been appointed superintendent nurse. She was trained at Salford Union Infirmary, Manchester, and was afterwards charge sister at Wolstanton and Burslem Union Infirmary and at Uckfield Union Infirmary, and head nurse and Queen's nurse, Salford.

KILSYTH FEVER HOSPITAL.—Miss Marian Orr has been appointed nurse-matron. She was trained at Lightburn Hospital, Glasgow.

LEEDS TOWNSHIP INFIRMARY.—Miss Clara E. Quipp and Miss Mary Lindley have been appointed sisters. Miss Quipp was trained at Ecclesall Bierlow Infirmary, Sheffield. Miss Lindley was trained at the Leeds Township Infirmary.

BATLEY AND DISTRICT HOSPITAL, YORKS.—The Misses L. G. Render and Celia Ramplin have been appointed sisters. They were trained at the Leeds Township Infirmary and hold certificates for invalid cookery.

**NOTE.—Particulars of Appointments for insertion in this column will be welcomed.**

### Frauds on Soldiers.

ATTENTION has already been directed in THE HOSPITAL to frauds perpetrated on soldiers by advertisers of worthless foodstuffs and quack medicines which the relatives of men on active service are persuaded by glowing advertisements to purchase. A striking example of this kind of fraud is given by the Paris correspondent of the official organ of the American Medical Association. He relates that a certain firm manufactured meat pastilles for soldiers in the field, the advantages of which, they said, were as follows : "The strength of our meat juice is such that eight of these pastilles represent the nutritive value of 400 gm. of pure meat, or the daily nourishment of a man. . . . One box of twenty-four pastilles represents, in its small compass, a reserve of provisions for three days. For the soldier in the field, therefore, it is the indispensable aid which will sustain his strength during days of great fatigue, and in certain cases may save his life." The box of twenty-four pastilles is sold for 4.50 francs, and the box of twelve pastilles for 2.50 francs. One of the members of the firm has been prosecuted for fraud, and fined 1,000 francs, on the ground that this meat juice is incapable of sustaining life, is no more stimulant than the usual commercial extract of beef, and represents in nutritive value about its own weight of fresh meat, so that eight pastilles, weighing 20 gm., represents 20 gm. of fresh meat, and not 400 gm. as the advertisement indicates. It seems strange that in our own country the police should afford so little protection to the public against unscrupulous and unpatriotic advertisers who make grossly exaggerated claims for concentrated foodstuffs and quack medicines, sold at exorbitant prices and intended as gifts for soldiers. A study of some of the advertisements regularly appearing in the Press should suggest ample scope for action.

Rates of Subscription: Twelve months, 6/6; Six months, 4/-; Three months, 2/-, post free to any address in the United Kingdom. Abroad, Twelve months, 8/8; Six months, 5/-; Three months, 2/6, post free.—Address The Subscription Dept., "The Hospital," The Hospital Building, 28/29 Southampton Street, Strand, London, W.C.

